# Robust Diffeomorphic Mapping via Geodesically Controlled Active Shapes

**DOI:** 10.1155/2013/205494

**Published:** 2013-04-03

**Authors:** Daniel J. Tward, Jun Ma, Michael I. Miller, Laurent Younes

**Affiliations:** ^1^Department of Biomedical Engineering, Johns Hopkins University, Baltimore, MD 21218, USA; ^2^Siemens Healthcare, Hoffman Estates, Chicago, IL 60192, USA; ^3^Center for Imaging Science, Johns Hopkins University, Baltimore, MD 21218, USA

## Abstract

This paper presents recent advances in the use of diffeomorphic active shapes which incorporate the conservation laws of large deformation diffeomorphic metric mapping. The equations of evolution satisfying the conservation law are geodesics under the diffeomorphism metric and therefore termed geodesically controlled diffeomorphic active shapes (GDAS). Our principal application in this paper is on robust diffeomorphic mapping methods based on parameterized surface representations of subcortical template structures. Our parametrization of the GDAS evolution is via the initial momentum representation in the tangent space of the template surface. The dimension of this representation is constrained using principal component analysis generated from training samples. In this work, we seek to use template surfaces to generate segmentations of the hippocampus with three data attachment terms: surface matching, landmark matching, and inside-outside modeling from grayscale T1 MR imaging data. This is formulated as an energy minimization problem, where energy describes shape variability and data attachment accuracy, and we derive a variational solution. A gradient descent strategy is employed in the numerical optimization. For the landmark matching case, we demonstrate the robustness of this algorithm as applied to the workflow of a large neuroanatomical study by comparing to an existing diffeomorphic landmark matching algorithm.

## 1. Introduction

There have been many approaches to segmentation in medical imaging, including both the active shape methods pioneered by Kass et al. [1] and template based approaches pioneered by Dann et al. [2]. For studying images made up of simple homogeneous structures such as anatomical structures, local active evolution methods [1, 3–5] which are encoded through their boundary representations are natural. In such methods, the complexity of the representation is reduced from an encoding based on the dimension of the extrinsic background space containing the object, to the dimension of the boundary.

Given the line of work in template based computational anatomy which has emphasized the important role of diffeomorphisms for defining bijective correspondence between coordinate systems, it is natural to constrain the iterative methods of active shapes so that shape evolution preserves the original topology of the template. This is the intention of the diffeomorphic active contour (DAC) approaches taken by Younes et al. [6–8], including in the local evolution equations the diffeomorphism constraint. DAC methods, in a form similar to the original methods of Christensen et al. and Trouvé [9, 10], only optimize for the final position of the deformable template and not for the evolution process that leads to it. The approach adopted herein results in an entire trajectory through shape space, allowing basic prior knowledge, that is, proximity to a template, to be incorporated in the estimate of a shape.

The trajectories considered are geodesic flows, which are deduced from the Riemannian structure associated to large deformation diffeomorphic metric mapping (LDDMM) [10–12]. Geodesics are characterized by a conservation law [7, 13–16] on the “momentum” associated to the evolution, where we describe in Section 2 what is meant by momentum in this context. This allows a further reduction in complexity from a time varying flow to a single initial condition: an initial momentum vector. In other words, a target shape is represented as the endpoint of a geodesic flow from a template and can be encoded by one such vector. In this setting, knowledge of shape variability is straightforwardly incorporated via prior distributions on initial momentum. We call these connections geodesically controlled diffeomorphic active shapes (GDAS).

In this paper, we examine robust LDDMM via geodesically controlled diffeomorphic active shape models. The GDAS method allows us to introduce prior distributions so as to support the diffeomorphic large deformations of unconstrained LDDMM (taking advantage of the reduction in complexity from a time varying flow to an initial condition), while at the same time constraining the mapping, so it is indexed to neuroanatomical shapes such as the subcortical structures (taking advantage of the reduction in complexity from background space to structure boundaries). We demonstrate that these mappings are robust to small variations associated with the MRI measures of the structures. This is accomplished by constraining the initial momentum of the GDAS solutions to be in the span of a finite-dimensional basis constructed from PCA associated with large-scale surface-based [17] anatomical studies and by penalizing our initial momentum estimates in basis directions of low variability as recently also derived in Qiu et al.'s work [18].

As with the classical active shape methods (described for example in [19–25], we pose the GDAS problem in the variational setting with the data term used for matching derived from various representations of a partition of the “scenes” including (i) collection of structures defined via triangulated meshes, (ii) a collection of structures defined through feature points, and (iii) a collection of homogeneous structures defined via inside-outside appearance model [26–30]. In case (iii), the process is iteratively driven by a voxel's likelihood of being interior or exterior of the region of interest (ROI), with the shape controlled by the conservation law geodesic dynamics. In our model, the appearance likelihood at each voxel only depends on whether the voxel is inside or outside the surface. It is estimated from the MRI training samples and modelled as Gaussian mixtures [31, 32] learned with the expectation maximization algorithm.

Vailliant et al. [33] first proposed this framework in a discussion of statistics on diffeomorphisms, and more recently Qiu et al. [18] addressed this problem and derived an algorithm for the case of surface-to-surface matching. Here, we consider a general data attachment term and provide a variational solution. We develop and implement a gradient descent algorithm for the case of surface matching, landmark matching, and grayscale image segmentation. A lack of robustness is an important challenge to high dimensional registration and a barrier to its automation and use in high throughput studies. We show that GDAS provides an efficient method for constraining LDDMM to incorporate the finite-dimensionality of typical shape variation and emphasize the robust performance of our methods on challenging biological datasets

## 2. Geodesic Diffeomorphic Evolution for Active Surfaces

In our region-of-interest (ROI) approaches to subcortical structure analysis in the human brain, our goal is to robustly segment anatomical structures (in particular neuroanatomical structures such as the hippocampus, caudate nucleus, etc.) from the surrounding environment using a given set of data (such as manually placed landmarks within an ROI, coarse segmentations, or MR images). Typically, anatomical structures have their own characteristic shapes and appearance which must be learned from training data to successfully perform segmentation.

### 2.1. Conservation Law Controlled Diffeomorphic Evolution

The methodology of tangent space representation has been a powerful tool in computational anatomy, since it was proposed in [16]. In this context, evolution of visual structures, like points, curves, surfaces, and images is governed by geodesic equations. By the law of momentum conservation, the initial state of the equations determines the entire trajectory of evolution and can be used as a representation of the trajectory endpoint. We refer to [16, 34] for more details and context in shape spaces modeled as homogeneous spaces under diffeomorphic action and describe here a special form of the associated equations that will be adapted to our needs.

For a triangulated surface *S*_0_ in ℝ^3^ with vertices *x*_1_,…, *x*_*L*_, the initial momentum *ρ*(0) : *S*_0_ → ℝ^3^ can be represented by a vector *a*_*l*_ at each vertex through *ρ*(0) = ∑_*l*=1_^*L*^ *a*_*l*_ *δ*(*x*_*l*_). One can derive the geodesic equation for the evolution of *S*_0_, which is equivalent to the geodesic equation for point sets [16]. We define a radially symmetric smoothing kernel *K* on ℝ^3^ × ℝ^3^. A typical choice, used here, is

(1) $$\begin{array}{c} K\left(x,y\right)=\exp\left(-\frac{{||x-y||}^{2}}{2\tau^{2}}\right). \end{array}$$

With *K*(*x*, *y*) = *γ*(||*x* − *y*||^2^), we denote *γ*_*kl*_ = *γ*(||*x*_*k*_ − *x*_*l*_||^2^), and *γ*_*kl*_′ = *γ*′(||*x*_*k*_ − *x*_*l*_||^2^). The geodesic evolution satisfies

(2) $$\begin{array}{c} \frac{\text{d}x_{k}}{\text{d}t}=\sum_{l=1}^{L}\gamma_{kl}a_{l},\\ \frac{\text{d}a_{k}}{\text{d}t}=-2\sum_{l=1}^{L}\gamma_{kl}^{\prime }\left(a_{l}\cdot a_{k}\right)\left(x_{k}-x_{l}\right), \end{array}$$

where the notation *a* · *b* refers to the usual dot product between vectors in ℝ^3^. Once the initial position of the vertices, *x*(0) = (*x*_1_(0),…, *x*_*L*_(0)), and the *initial momentum vector*, *α*(0) = (*a*_1_(0),…, *a*_*L*_(0)), are provided, the evolution of the point set is uniquely determined. The endpoints of the evolution *x*(1) = (*x*_1_(1), *x*_2_(1),…, *x*_*L*_(1)) correspond to the deformed surface *S*(1). It can be shown that (2) has solutions over arbitrary time intervals [34]. Equation (2) induces a diffeomorphism, *ϕ*, that interpolates the evolution of the vertices via the equation

(3) $$\begin{array}{c} \frac{\text{d}\phi }{\text{d}t}\left(t,x\right)=\sum_{l=1}^{L}K\left(\phi \left(t,x\right),x_{l}\right)a_{l}. \end{array}$$

The conservation law associated to (2) then takes the form *a*_*l*_(*t*) = *Dϕ*(*t*, *x*_*l*_)^−*T*^ *a*_*l*_(0).

Hence, we are able to represent a shape by an initial momentum vector (as opposed to a function or distribution) and a template. Furthermore, the variability of shapes can be described from probabilistic properties of initial momentum. This is the basic representation for the tangent space PCA of shapes.

### 2.2. Finite Dimensional Representation via PCA in the Tangent Space

Clearly, as the discretization of the surface gets fine, the dimension of the control point representation of the momentum goes to infinity. We want to characterize the main modes of statistical variations in our data, of which there is a small number up to some acceptable error. This enables us to write the momentum vector in a finite dimensional span regardless of the discretization.

It is natural to do this empirically based on training samples. PCA depends on the definition of an inner product in the considered space. In our context, the inner product derives from the Riemannian metric that led to the geodesic equation. Details of corresponding PCA have been described previously [33].

We calculate a population template *T* using the approach in [35], but several other algorithms are available [36–38]. We denote the inner product between two initial momentum vectors *α* and $\tilde{\alpha }$, as ${\langle \alpha ,\tilde{\alpha }\rangle }_{T}$. Assume we have recovered mean initial momentum vector $\overset{-}{\alpha }$, *D* orthonormal prinicipal components *u*_*i*_ (with 〈*u*_*i*_, *u*_*j*_〉_*T*_ = *δ*_*ij*_), and an estimate of variance along each component *λ*_*i*_. We note that any initial momentum vector *α* can be projected onto the principal space as

(4) $$\begin{array}{c} \text{proj}\left(\alpha \right)=\overset{-}{\alpha }+\sum_{i=1}^{D}k_{i}u_{i},\;\text{where}\;\;k_{i}={\langle \alpha -\overset{-}{\alpha },u_{i}\rangle }_{T}. \end{array}$$

By parameterizing our deformations with respect to the *k*_*i*_, the variability of the surface shape is constrained by the principal space generated from the training set.

## 3. Segmentation Algorithms

### 3.1. Variational Formulation of Volumetric Segmentation

We formulate segmentation problems within an energy minimizing scheme. The energy includes two terms: one term is to constrain the shape in the principal space, with appropriate weights derived from PCA, and the other term regulates the error of mismatch, which we will define in a general sense here and show specific examples in Sections 3.2 to 3.4.

We introduce some notation. Let *Ω* ⊂ ℝ^3^ be the background space. Let *T* denote the template, which will be assumed to be a closed surface. After learning, the selected principal space of initial momentum is spanned by *D* orthonormal vectors *u*_1_, *u*_2_,…, *u*_*D*_ corresponding to decreasing eigenvalues *λ*_1_, *λ*_2_,…, *λ*_*D*_.

Our goal is to segment the region-of-interest (ROI) from *Ω* using a deformable model; that is, we want to deform the template *T*, so that it overlaps with the boundary of the ROI. We assume that *T* is a triangulated surface, with vertex set *x* ^0^ = (*x*_1_^0^,…, *x*_*L*_^0^). For an initial momentum *α*, we let *x* ^*α*^ denote the solution at time *t* = 1 of (2) initialized with *x*_*k*_(0) = *x*_*k*_^0^ and *a*_*k*_(0) = *α*. We let *S* ^*α*^ denote the triangulated surface that inherits the topology of *T* with displaced vertices *x* ^*α*^.

We now describe the two terms in our energy that balance the prior knowledge we have of the diffeomorphic deformation with the accuracy of the segmentation. The initial momentum *α* is constrained to the principal space and takes the form $\alpha \;(0)=\overset{-}{\alpha }+\sum_{n=1}^{D}k_{n}u_{n}$. This prior knowledge is regulated by the coefficients of principal components, scaled by eigenvalues, resulting in the first term of our cost function:

(5) $$\begin{array}{c} E_{1}=\sum_{n=1}^{D}\frac{k_{n}^{2}}{\lambda_{n}}. \end{array}$$

We define the accuracy of segmentation based on some function *E*_2_ of only available data (e.g., an image, a set of landmarks, or a surface) and the configuration of our deformed template *S* ^*α*^. For the time being, we assume that the derivative of this function with respect to each of the *x*_*i*_^*α*^ is known, and we denote the vector of this derivative by ∂*E*_2_/∂*x* ^*α*^.

Our goal is to find the optimal coefficients *k*_1_, *k*_2_,…, *k*_*D*_ that minimize the energy:

(6) $$\begin{array}{c} E=E_{1}+\frac{1}{\sigma^{2}}E_{2}. \end{array}$$

The variational problem is solved by calculating the derivative of *E* with respect to each coefficient *k*_*n*_. The derivative of *E*_1_ is trivial:

(7) $$\begin{array}{c} \frac{\partial E_{1}}{\partial k_{n}}=\frac{2k_{n}}{\lambda_{n}}. \end{array}$$

The derivative of *E*_2_ can be calculated by the chain rule

(8) ∂E2∂kn=(∂E2∂xα)T(∂xα∂α)un=(∂E2∂α)Tun.

While the term ∂*x* ^*α*^/∂*α* is unknown, its product with ∂*E*_2_/∂*x* ^*α*^ (which we denote as ∂*E*_2_/∂*α*) can be calculated numerically by solving a system of linear ordinary differential equations. This adjoint method has become common in this context and is derived, for example, in [35]. It is described for completeness in Appendix B. The derivative of *E* is given by

(9) $$\begin{array}{c} \frac{\partial E}{\partial k_{n}}=\frac{2k_{n}}{\lambda_{n}}+\frac{1}{\sigma^{2}}{\left(\frac{\partial E_{2}}{\partial \alpha }\right)}^{T}u_{n}. \end{array}$$

We now discuss three applications and explain examples of *E*_2_ and their gradients.

### 3.2. Robust Surface Matching

In challenging surface mapping applications, it can be necessary to regularize the mappings to avoid undesirable results, and GDAS provides a powerful method for doing so.

In particular, volumetric segmentations of neuroanatomy are often readily available. Converting them to an isosurface for analysis and display is standard, and GDAS provides a method to convert such an isosurface to one reflecting the typicality and variability of a population, rather than features of the volumetric data with an unnatural voxelized structure. Our goal here is to provide a tool to correct for such erroneous segmentations.

This application is essentially equivalent to that presented in [18]. We retain it here as it is the most natural application of GDAS (priors learned from surface matching are used to regularize surface matching), to develop a notation consistent with that for our other applications and to demonstrate robust performance on poorly behaved datasets.

#### 3.2.1. Notation

The deformed template surface *S* ^*α*^ is triangulated with vertices *x*_1_^*α*^,…, *x*_*L*_^*α*^. Suppose the template surface has *M* faces denoted as *F* = (*f*_1_,…, *f*_*M*_). Each face is represented by an ordered triple of vertices: *f* = (*x*_*f*,1_^*α*^, *x*_*f*,2_^*α*^, *x*_*f*,3_^*α*^). We define oriented edges on face *f* by

(10) $$\begin{array}{c} e_{f,1}^{\alpha }=x_{f,2}^{\alpha }-x_{f,3}^{\alpha },\\ e_{f,2}^{\alpha }=x_{f,3}^{\alpha }-x_{f,1}^{\alpha },\\ e_{f,3}^{\alpha }=x_{f,1}^{\alpha }-x_{f,2}^{\alpha }, \end{array}$$

the area-weighted normal to face *f* by

(11) $$\begin{array}{c} N_{f}^{\alpha }=\frac{1}{2}e_{f,3}^{\alpha }\times e_{f,2}^{\alpha }, \end{array}$$

and the face center by

(12) $$\begin{array}{c} c_{f}^{\alpha }=\frac{1}{3}\left(x_{f,1}^{\alpha }+x_{f,2}^{\alpha }+x_{f,3}^{\alpha }\right). \end{array}$$

Similarly, suppose the target surface has *L*′ vertices *y*_1_,…, *y*_*L*_ and *M*′ faces denoted as *F*′ = (*f*_1_′,…, *f*_*M*′_′), with oriented edges *e*_*f*′,1_′, *e*_*f*′,2_′, *e*_*f*′,3_′, area-weighted normals given by *N*_*f*′_′, and face center *c*_*f*′_′.

Similarly to the case for velocity fields, we define a smoothing kernel *K*_*S*_ (*S* for “surface”) of the form in (1) to be used for comparing two surfaces.

#### 3.2.2. Energy

Following [17], we embed surface matching in a more general “current matching” problem. This results in an energy to be minimized taking into account closeness between two surfaces, as well as orientation of normals:

(13) E2=∑i=1M∑ j=1MNiαTKS(ciα,cjα)Njα −2∑i=1M∑ j=1M′NiαTKS(ciα,cj′)Nj′ +∑i=1M′∑ j=1M′Ni′TKS(ci′,cj′)Nj′.

The constant *σ* ^2^ in (6) is determined heuristically.

#### 3.2.3. Energy Gradient

We refer the reader to [17] for the derivation of this energy gradient and present the result only here:

(14) ∂E2∂xα|k=∑f:xkα=xf,iα[∑j=1Mef,iα×KS(cjα,cfα)Njα      +23(NfαT∂∂cfαKS(cjα,cfα))Njα      −∑j=1M′ef,iα×KS(cj′,cfα)Nj′      −23(NfαT∂∂cfαKS(cj′,cfα))Nj′],

where the first sum is over faces *f* for which *x*_*k*_^*α*^ is a vertex, and the symbol *i* is reserved for the index of that vertex on each such face (*i* ∈ {1,2, 3}).

### 3.3. Robust Landmark Matching

A further application of our framework involves ROI analysis methods based on diffeomorphic landmark matching. Given a template surface containing *K* landmarks located on vertices, a trained technician places corresponding landmarks in T1 MR images. Diffeomorphic landmark matching provides a segmentation of the structure of interest in each T1 image by applying the landmark-based transformation to the entire template surface. This procedure is advantageous, because it provides a compromise between the speed of automatic segmentation and the accuracy of hand segmentation.

However, variability in landmark placement and sparsity of landmarks can occasionally lead to unsatisfactory segmentation results and to a time-consuming quality control stage where such segmentations are fixed manually. We propose to regularize the problem, taking into account landmark placement variability based on voxel size, as well as shape variability learned from PCA.

#### 3.3.1. Notation

For simplicity, we assume that landmarks on the template are chosen among the vertices of its representation as a triangulated surface. We denote by *X*_*i*_(*t*) = *x*_*l*(*i*)_(*t*) the *i*th landmark, which is placed on a template vertex *l*(*i*) and flowed according to (2) up to time *t*. Therefore, *X*_*i*_(1) = *x*_*l*(*i*)_^*α*^. We define the corresponding landmark placed on the target image as *Y*_*i*_.

#### 3.3.2. Energy

We assume that the only available information on the targets is landmarks at positions homologous to those on the template. The accuracy of segmentation is then based on the squared distance between deformed template and target landmarks, leading to the term

(15) $$\begin{array}{c} E_{2}=\sum_{i=1}^{K}{||X_{i}\left(1\right)-Y_{i}||}^{2}. \end{array}$$

The weighting parameter is chosen on the order of voxel size *σ* ^2^ = ((Δ*x*/2)^2^ + (Δ*y*/2)^2^ + (Δ*z*/2)^2^)/3 (equal to 0.4475 for our application) where Δ*x*, Δ*y*, and Δ*z* are the target image's voxel dimension.

#### 3.3.3. Energy Gradient

The variation of *E*_2_ with respect to the *k*th deformed template vertex is 0 if this vertex does not correspond to a landmark, and 2(*X*_*k*_ − *Y*_*k*_) = 2(*x*_*l*(*k*)_^*α*^ − *Y*_*k*_) if it does. That is,

(16) $$\begin{array}{c} {\frac{\partial E_{2}}{\partial x_{\alpha }}|}_{k}=\{\begin{array}{cc} 0 & k\;\text{is}\;\text{not}\;\text{a}\;\text{landmark},\\ 2\left(x_{k}^{\alpha }-Y_{j}\right) & k\;\text{is}\;\text{the}\;j\text{th}\;\text{landmark}. \end{array} \end{array}$$

### 3.4. Robust Image Segmentation

We seek to automatically segment subcortical structures from MR images. For simplicity, we assume that such structures are relatively homogenous throughout and therefore chose an appearance model for voxel intensities that depend on location only through whether they are inside the structure or not. To perform image segmentation, we seek to partition the space into high integrated voxel-likelihood under such an inside-outside model. The approach can be generalized to more complex appearance models (involving higher order image features, e.g.) in a straightforward way.

#### 3.4.1. Appearance Model

Gaussian mixtures are widely employed to model voxel intensity of medical imaging. That is, a p.d.f of intensity *I* at a certain location in the tissue is in the form of

(17) $$\begin{array}{c} p\left(I;x\right)=\sum_{q=1}^{Q}\pi_{q}\left(x\right)\frac{1}{\sqrt{2\pi \sigma_{q}^{2}\left(x\right)}}\exp\left(-\frac{{\left(I-\mu_{q}\left(x\right)\right)}^{2}}{2\sigma_{q}^{2}\left(x\right)}\right), \end{array}$$

where *π*_*q*_, *μ*_*q*_, and  *σ*_*q*_^2^ denote the weight, mean and variance of *q*th (out of *Q*) Gaussian component, respectively.

In our work, we assume that the intensities at all points of the interior region (resp., exterior region) of the surfaces share the same mixed Gaussian distribution and the p.d.f's are denoted as *p*_int⁡_ and *p*_ext_, respectively.

Given the number of mixture components, the maximum likelihood estimator for the parameters can be computed using the EM algorithm [39]. Our estimation of *p*_int⁡_ and *p*_ext_ (using mixtures of Gaussians) is performed on the basis of training images with manual segmentation, in which the collection of all intensity values of voxels inside (resp., outside) the ROI are used for *p*_int⁡_ (resp., *p*_ext_).

#### 3.4.2. Energy

We define the accuracy of segmentation using integrals of likelihood of being misclassified, and we define the mismatch:

(18) E2=∫x∈int⁡(S)log⁡(pext(I(x)))dx +∫x∈ext(S)log⁡(pint⁡(I(x)))dx,

where we denote the interior and exterior of a closed surface *S* by int⁡(*S*) and ext(*S*), respectively.

The constant *σ* ^2^ in (6) is determined heuristically.

#### 3.4.3. Energy Gradient

The energy gradient is derived in Theorem A.1 in Appendix A. With *g*(*y*) = log⁡  [*p*_ext_(*y*)/*p*_int⁡_(*I*(*y*))] and *m*_*f*,*i*_^*α*^ the midpoint of the *i*th edge of face *f*, we have

(19) $$\begin{array}{c} {\frac{\partial E_{2}}{\partial x^{\alpha }}|}_{k}=\sum_{f:x_{k}^{\alpha }=x_{f,i}^{\alpha }}\frac{1}{2\left|N_{f}\right|}\int_{f}g\left(y\right)\left(m_{f,i}^{\alpha }-y\right)\times e_{f,i}^{\alpha }\;d\sigma_{f}. \end{array}$$

### 3.5. Numerical Implementation

We use gradient descent to optimize the PCA coefficients and iteratively update *k*_*n*_ with *k*_*n*_ − *ϵ*(∂*E*/∂*k*_*n*_) until convergence. The discretization of the surface integrals in (19) is simply performed by replacing *g*(*y*)(*m*_*f*,*i*_^*α*^ − *y*) × *e*_*f*,*i*_^*α*^ by its value at *y* = *c*_*f*_^*α*^, the center of face *f*, with

(20) $$\begin{array}{c} \frac{1}{2}\left(m_{k,f}^{\alpha }-c_{f}^{\alpha }\right)\times e_{k,f}^{\alpha }=\frac{1}{3}N_{f}^{\alpha }. \end{array}$$

This yields the approximation

(21) $$\begin{array}{c} {\frac{\partial E_{2}}{\partial x^{\alpha }}|}_{k}=\frac{1}{3}\sum_{f:x_{k}\in f}g\left(c_{f}^{\alpha }\right)N_{f}^{\alpha }, \end{array}$$

that has been used in our implementation.

We summarize these steps in Algorithm 1. The complete procedure, including training, is summarized in Algorithm 2.

## 4. Experimental Methods

To demonstrate the proposed algorithm, for each of the three data attachment terms, we use data being processed as part of many of our region-of-interest (ROI) biological studies in schizophrenia, depression, Alzheimer's disease, ADHD, and autism [40–45] (for landmark matching and image segmentation) and Alzheimer's Disease Neuroimaging Initiative (ADNI) study (for surface matching and PCA training). Shown in the accompanying figures are 5 examples demonstrating the robustness constraints imposed by performing large deformation mapping in the span of the first few PCA dimensions learned from our empirical mappings. For the case of landmark matching, we have integrated it into the workflow of several large neuroanatomical studies (e.g., [46]). We therefore include this application as a case study, quantifying performance in detail and demonstrating improvement we expect to gain. Our hypothesis when beginning this work was that the GDAS algorithm would exhibit increased robustness compared to more standard methods, without significantly sacrificing accuracy.

Data used in the preparation of this paper were obtained from the ADNI database (http://adni.loni.ucla.edu/). The ADNI was launched in 2003 by the National Institute on Aging (NIA), the National Institute of Biomedical Imaging and Bioengineering (NIBIB), the Food and Drug Administration (FDA), private pharmaceutical companies, and nonprofit organizations, as a $60-million, 5-year public-private partnership. The primary goal of ADNI has been to test whether serial magnetic resonance imaging (MRI), positron emission tomography (PET), other biological markers, and clinical and neuropsychological assessment can be combined to measure the progression of mild cognitive impairment (MCI) and early Alzheimer's disease (AD). Determination of sensitive and specific markers of very early AD progression is intended to aid researchers and clinicians to develop new treatments and monitor their effectiveness, as well as lessen the time and cost of clinical trials.

The Principal Investigator of this initiative is Michael W. Weiner, MD, VA Medical Center and University of California San Francisco, CA, USA. ADNI is the result of efforts of many coinvestigators from a broad range of academic institutions and private corporations, and subjects have been recruited from over 50 sites across the US and Canada. The initial goal of ADNI was to recruit 800 adults, ages 55 to 90, to participate in the research, approximately 200 cognitively normal older individuals to be followed for 3 years, 400 people with MCI to be followed for 3 years, and 200 people with early AD to be followed for 2 years. For up-to-date information, see http://www.adni-info.org/.

### 4.1. Principal Component Analysis

Given a template and a set of target surfaces (650 for the ADNI study), we perform principal component analysis on initial momentum data as described in Section 2.2. The same ADNI template and PCA model will be used in each of our applications, even for data taken from other datasets. We plot the variance as a function of number of dimensions and identify the number of dimensions required to account for 95% of the variance.

### 4.2. Surface Matching Study

**Figure 1:**
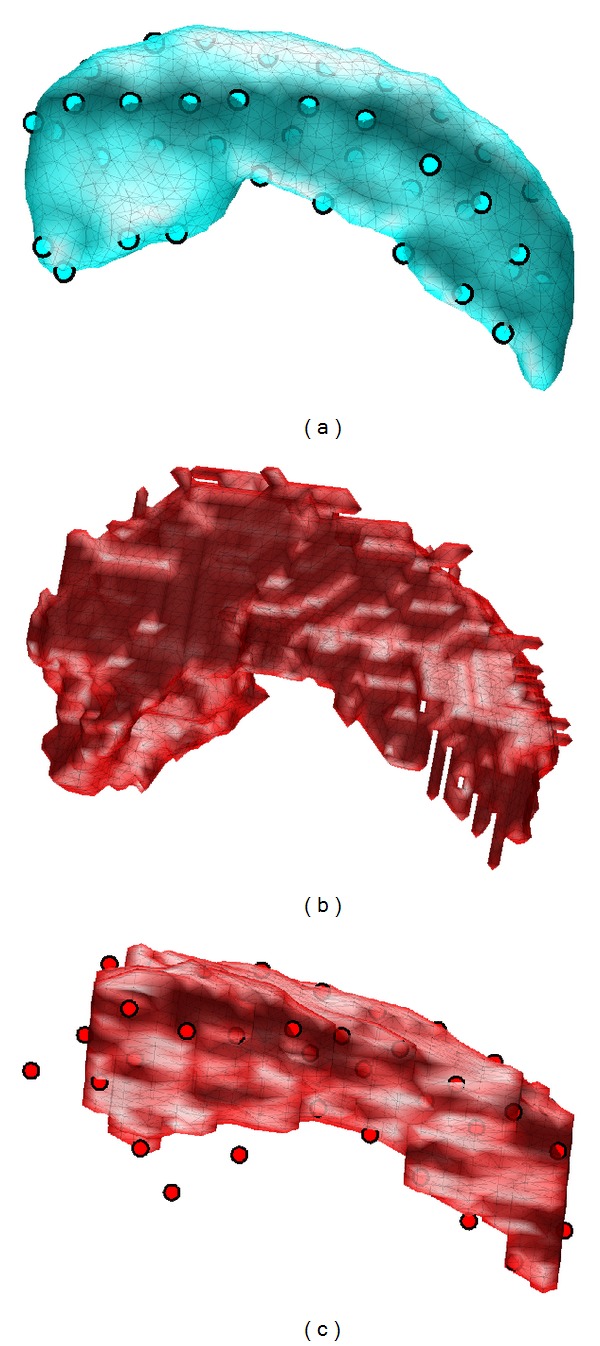
(a) Template hippocampus for ADNI dataset. (b) Hippocampus isosurface from example volumetric parcellation. (c) Isosurface of example hippocampus manual segmentation for our landmark datasets.

As part of the ADNI study, volumetric parcellations (performed using Freesurfer, described, e.g., in [47]) of whole brains are available at a series of time points. The *t* = 0 data has been studied and a template (see Figure 1(a)) as well as a population of initial momenta data has been calculated [48]. To study their changing shapes over time, we wish to convert such binary segmentations to surfaces. However, the voxelized nature of the segmentations makes simple isosurfaces unacceptable (as shown in Figure 1(b)).

We therefore employ the technique of matching our template to such an isosurface, using the constraints of a smooth deformation regularized by PCA to avoid the unnatural appearance of the isosurface. We show example results from our GDAS surface matching algorithm, and compare them to typical results from the traditional surface matching LDDMM algorithm.

### 4.3. Landmark Matching Study

Our segmentation pipeline for our ROI methods is described in [40–44, 46]. The relevant portion (the landmark matching phase) for one such study is summarized here. Thirty-eight landmarks are placed along the left and right hippocampi in 441 0.93  ×  0.93  ×  2.0 mm T1 images of the brain. The first was placed at the tip of the head of the hippocampus (the center of the most anterior slice containing the hippocampus in a T1 image), and the second was placed at the tip of the tail of the hippocampus (the center of the most posterior slice containing the hippocampus). The distance between these two was then divided into 9 slices from anterior to posterior, and on each slice 4 landmarks were placed at the superior, inferior, medial, and lateral margins of the hippocampus. This manual procedure takes approximately 10 minutes for a trained technician to complete, as compared to over 2 hours for a full hand segmentation of images of this size.

In the existing segmentation and analysis pipeline, a template surface was chosen as the left hippocampus for a single subject, and a manual segmentation and resulting isosurface were generated for this case. After a similitude alignment (including reflecting right hippocampi to match left) landmark LDDMM [49] was used to map this template to each target, defining a segmentation surface and binary image for each patient.

However, this procedure was found to suffer from lack of robustness, and roughly 30 out of 441 cases were unacceptable. A laborious phase of quality control was necessary involving identifying problematic or distorted segmentations, manually editing their binary images, and regenerating isosurfaces.

To measure whether our prior model provides enough robustness to avoid such issues, we chose 5 challenging cases of left hippocampi (as identified during quality control inspection), where manual intervention was required and 11 typical cases, and we examined the performance improvement using the proposed algorithm rather than that outlined above. These cases were manually segmented by a trained technician to provide a gold standard, and associated isosurfaces were generated for further evaluation. Furthermore, for 3 cases requiring intervention and 2 typical cases (those illustrated in Figure 4), a second manual segmentation was obtained to give a sense of interrater variability.

Note that the segmentations that are shown here do not constitute the final output of the ROI pipeline described in [40], in which they would be further processed. That is, the results of standard landmark mapping shown here are not reflective of the final segmentations. However, we expect improvement at this stage to contribute to overall improvement.

The template with its associated landmarks is shown in Figure 1(a). In Figure 1(c), an example isosurface generated from a manual segmentation is shown together with its associated landmarks. Note that landmarks were placed on template surface vertices, but the target landmarks were placed independently (by a different technician) from the gold standard manual segmentation.  Figure 1 shows the uncertainty of landmark placement, particularly in the region of the hippocampus' head and demonstrates the need to include landmark placement variability in the segmentation algorithm. In these figures and throughout this paper, the color cyan will be used for the template, red for the target, blue for our new results, and green for results using existing algorithms.

### 4.4. Image Segmentation Study

To demonstrate the capabilities of the GDAS image segmentation algorithm, 5 examples for the same dataset as the landmark matching study are shown. We anticipate that good initial alignment will be important for high quality segmentations, and so the same landmark based similitude registration as above will be used to initialize the target data in this study.

We use 4 outside and 3 inside components for our Gaussian mixture model. The mixture model is trained based on gold standard segmentations from the remaining cases in a “leave-one-out” fashion. A histogram equalization intensity transformation is applied to each T1 image to match the first training sample, based on data from a neighborhood (±5 voxels) around the landmarks, before estimating mixture model coefficients. A similar histogram equalization is applied to the target image (to match the first training sample) before beginning the segmentation process.

### 4.5. Analysis Methods

In analyzing results, we seek to demonstrate two main ideas. First, the accuracy of GDAS is comparable to existing methods, and second its robustness is improved. Accuracy is demonstrated using three techniques. First, we use visual inspection. Second, *κ* scores [50] are used to compare our results to gold standard segmentations. This score is defined by

(22) $$\begin{array}{c} \kappa =\frac{p_{\text{agree}}-p_{\text{random}}}{1-p_{\text{random}}}, \end{array}$$

where *p*_agree_ is the fraction of voxels in which the given segmentation agrees with the manual segmentation, and *p*_random_ is the fraction you would expect by random chance (based only on the volumes of foreground and background). We calculate *κ* scores using a Monte-Carlo method, generating uniformly distributed points and checking if they are inside one or both segmentations. For applications involving subcortical structures, a value of *κ* = 0.8 is generally considered quite good. Third, surface-to-surface distance cumulative distribution functions (c.d.f.s) are used to quantify average proximity of surfaces. At each vertex on each surface, we calculate the distance to the nearest vertex on the other surface and analyze the distribution of these distances. We examine entire c.d.f.s and also examine thresholds: distance at which 50% of vertices are closer than and distance at which 80% of vertices are closer than.

Robustness is also demonstrated using visual inspection, with care taken to highlight challenging regions. Surface-to-surface distance histograms are also restricted to such regions, highlighting challenging areas rather than averaging over the entire surface. Additionally, we quantify surface smoothness by measuring integrated sum of squares of principal curvatures over the deformed template surface (this quantity is scale invariant); the assumption being that smoother surfaces more accurately reflect natural anatomy in these applications.

Further, to overcome limitations surrounding the accuracy of manual segmentation and to emphasize the robustness of our algorithm, we evaluate its performance on simulated data. Five hippocampal shapes are generated according to the PCA model in Section 4.1, and landmarks are placed on the vertices shown in Figure 1(a) with additive Gaussian noise of variance 0.01, 0.1, 1, 10, and 100 times that of the weighting parameter discussed in Section 3.3.2. Traditional LDDMM landmark matching as well as GDAS is run on this dataset using the same parameters as for our real data, emulating a scenario where errors in landmark placement are unknown beforehand. We demonstrate robustness by reporting *κ* scores as a function of landmark noise for both algorithms. Lastly, we show the accuracy at which PCA coefficients are recovered using Mahalanobis distance, and *P* values corresponding to such distances are shown for real and simulated data.

## 5. Results

### 5.1. Principal Component Analysis

**Figure 2:**
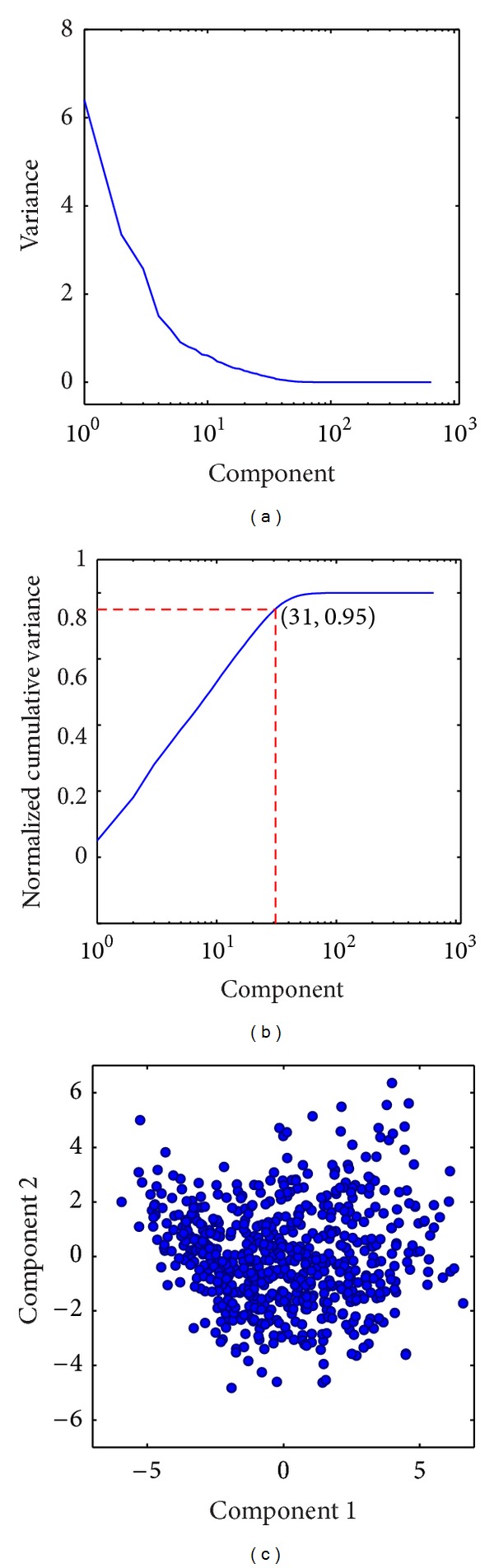
(a) The variance of each PCA coefficient in the left hippocampus ADNI population. (b) The normalized cumulative variance indicating the number of dimensions accounting for 95% of the variability. Note the semilog scale in (a) and (b). (c) A scatter plot of the first two PCA coefficients for this population.

After performing tangent space PCA with the left hippocampus for the ADNI dataset, we found 31 dimensions characterized 95% of the variability for this population (to be contrasted with 1184  ×  3 dimensions associated with a momentum vector at each vertex of the discretized surface). This is illustrated in Figure 2.

### 5.2. Examples: Surface Matching

**Figure 3:**
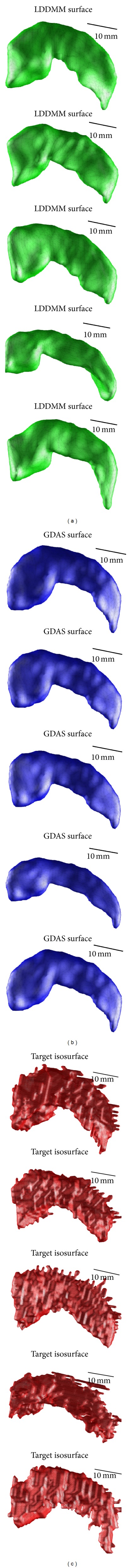
Examples of hippocampus surfaces resulting from using (left/green) surface LDDMM and (center/blue) GDAS surface matching. The target isosurface is shown at the right (red).

For 5 examples, the outcomes of traditional LDDMM surface matching [17] and GDAS surface matching are shown in Figure 3, with target isosurfaces shown on the right-hand side. Qualitatively speaking, the traditional LDDMM result tends to produce squared off hippocampal heads (left side in figure) due to outlier voxels, as well as an overestimation of the medial margin (bottom of figure) due to overfitting an outlier “ribbon” of voxels.

The constraints imposed in GDAS surface matching result in a useful and accurate segmentation reflective of the population being analyzed. The “fingerlike” and “ribbonlike” projections reflecting the voxelized structure of the target isosurface, as well as the set of constraints used in Freesurfer that are designed for an unrelated application, do not significantly influence the resulting surface.

### 5.3. Examples: Robust Landmark Matching

**Figure 4:**
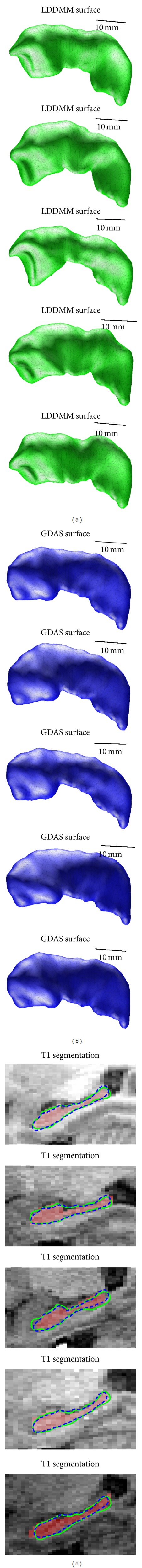
Segmentation results for standard landmark matching (left/green/solid) and GDAS landmark matching (center/blue/broken) for 5 examples (first 3 were identified for quality control; final 2 were not). Segmentations overlaid with corresponding T1 image and “ground truth” (red highlight) are shown in the right column.

For 3 cases requiring quality control and 2 typical cases, the outcome of landmark matching is shown in Figure 4. Traditional landmark matching is shown on the left side (green), while GDAS landmark matching is shown in the center column (blue). In the right-hand column, the surfaces are shown overlaid on a T1 image, with the gold standard segmentation shown in red.

Qualitatively, the improvement of the GDAS algorithm over traditional landmark matching is evident. Large distortions at the head of the hippocampus are common where landmark placement can be quite variable. Along the length of the hippocampus, deformations with scale characteristic of the distance between landmarking planes are easily seen. These issues are still common in those surfaces not requiring quality control. The GDAS algorithm avoids each of these pitfalls, avoiding overfitting landmarks while maintaining shape variability characteristic of the population.

**Figure 5:**
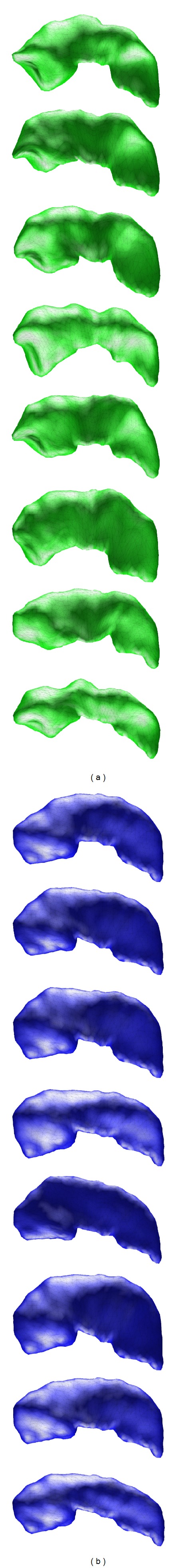
Segmentation results for standard landmark matching (left/green) and GDAS landmark matching (right/blue), for an additional 8 examples demonstrating GDAS overcoming common pitfalls.

Because poor performance of traditional landmark LDDMM motivated this analysis, we also display (in less detail) results for an additional 8 patients selected randomly from among those identified as performing poorly. These are shown in Figure 5 with traditional LDDMM results shown in the left column and GDAS results shown in the right column. It is evident that the distortions seen in the top three rows of Figure 4 are typical for this dataset, and that the GDAS results avoid these distortions in a similar manner.

### 5.4. Examples: Image Segmentation

An example of the results of Gaussian mixture modeling is shown as probability density functions in Figure 7. Measured data (after histogram equalization) is shown as a solid curve, and the results of mixture modeling as dashed curve. The Gaussian mixture parameters are quite similar in all cases examined. The “inside” region (narrow curve, blue and red) is a unimodal distribution describing subcortical gray matter. The challenge of this application can be seen from the “outside” region (broad curve, green and magenta), which is a more heterogenous mixture. It describes cerebrospinal fluid and white matter, as well as cortical gray matter and partial-volume voxels whose intensities are quite similar to the “inside”.

**Figure 6:**
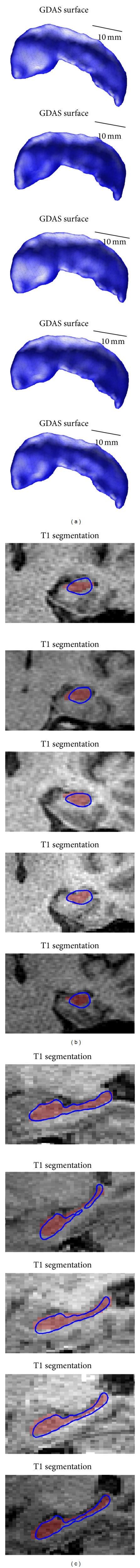
Example segmentations of T1 images using GDAS image segmentation based on inside-outside modelling. The resulting surfaces are shown on the left-hand side, and T1 images with gold standard (red highlight) and segmentation (blue curve) are shown in coronal (center) and sagittal (right) views.

**Figure 7:**
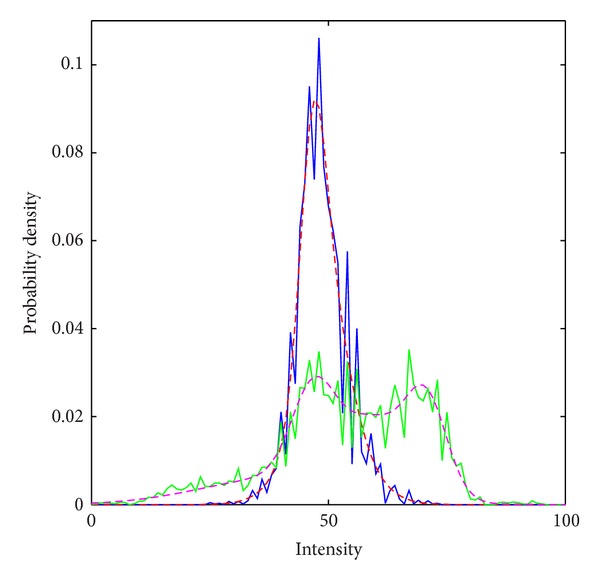
Mixture modeling is shown for inside (narrow curve, red/blue) and outside (broad curve, magenta/green) T1 voxel intensities (after histogram equalization). The T1 data is shown with broken lines and the mixture model with solid lines.

Five example segmentation results are shown in Figure 6. The performance appears satisfactory, an achievement considering the large overlap between inside and outside histograms seen in Figure 7. The PCA prior can prevent the template surface from deforming to erroneously include cortical gray matter in many cases, even though it is similar or identical in intensity to subcortical gray matter. This simple inside/outside model could likely be improved, for example, by including a heterogenous appearance model, or combining landmarks and intensity information in cost functions. However, this will be the subject of future research. The purpose of this section was to demonstrate the extensibility of the GDAS framework to a varied range of applications.

### 5.5. Evaluation: ROI Method Case Study

For the landmark matching application, we describe in detail the performance of the GDAS algorithm as compared to our existing method.

**Figure 8:**
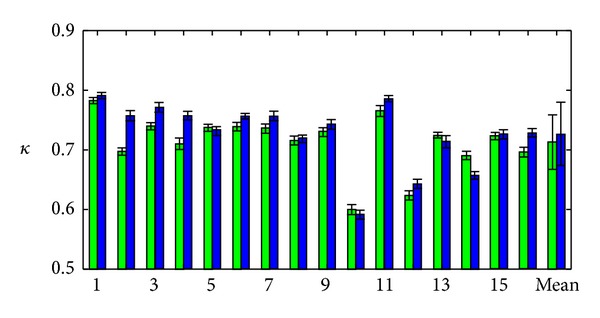
Kappa scores are shown for each of the 16 patients examined, with mean and standard deviation shown on the right. Green/left of pair: typical landmark matching; blue/right of pair: GDAS landmark matching.

The overlap on a large scale is quantified by *κ* scores, as shown in Figure 8 for each of the 16 test cases. The GDAS results tend to be similar, but better on average than those for landmark LDDMM. For typical landmark matching, the mean and standard deviation of *κ* is 0.7131 ± 0.0457, and for GDAS landmark matching, it is 0.7268 ± 0.0531. The difference is statistically significant (*P* < 0.05 in Student's paired *t*-test).

For those cases with two raters, we examine the second *κ* score, which differed from the first by 2.66% on average, to understand interrater variability. We present *κ* scores, averaged over the two raters in Table 1. In each case, the GDAS method performs superiorly for both raters, and this is reflected in the increased average *κ* scores from 0.732 to 0.751. Despite this improvement, it is interesting to note that the *κ* overlap between the two manual segmentations is comparable to that between the results of the two segmentation methods.

Examining overlap voxel by voxel, as in Figure 8, shows our algorithm making a small improvement in accuracy. However, the relatively larger improvement in robustness can be seen when examining surface shapes globally such as in Figures 4 and 5 and contrasting with expectations from knowledge of neuroanatomy. The region around the head was seen to be particularly challenging to segment in the traditional landmark case, and distortions occurring at the scale of landmark spacing give the impression that certain regions are “left behind.” To quantify accuracy globally, while acknowledging these specifically challenging areas, we use surface-to-surface-distance histograms and associated c.d.f.s.

**Figure 9:**
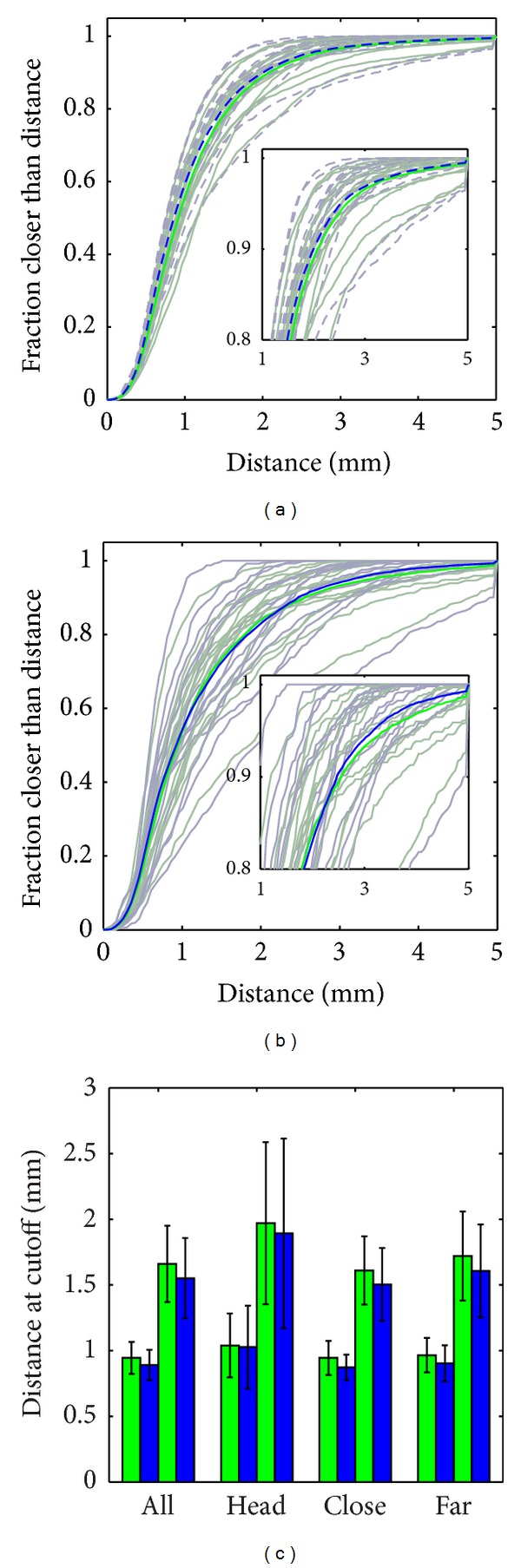
Surface-to-surface distance c.d.f.s including (a) all vertices and (b) only vertices within 10 mm of head landmark. Inset shows same data zoomed to ≥80%. Plot (c) shows the 50% (left pair in a set of four) and 80% (right pair in a set) crossing for the vertices shown in (a) (“All”) and (b) (“Head”), as well as within 2.5 mm of any landmark (“Close”) or not (“Far”). Green/solid/left of pair: traditional LDDMM; blue/broken/right of pair: GDAS.

These c.d.f.s are shown for all 16 patients (unsaturated colors, green: standard landmark matching, blue: GDAS) in Figure 9(a). Combining all vertices gives a single c.d.f indicative of the whole population (saturated colors). A CDF closer to the top left reflects a better segmentation. In Figure 9(b), we show the same analysis, but restricted to vertices within 10 mm of the head landmark. This analysis was repeated (not shown) with vertices restricted to those within 2.5 mm of any landmark, and those not within 2.5 mm of any landmark.

For each patient, the 50% and 80% crossings were measured and are plotted in Figure 9(c). In each set of four bars, the left two show 50% crossings, and the right two show 80% crossings. A smaller value indicates a better segmentation, but the 50% crossing indicates a “typical” region, while the 80% crossing indicates an “outlier” region. Our hypothesis was that the GDAS algorithm would show improvement in outlier regions, at the cost of poorer performance in typical regions. However, the data shows better performance from GDAS in all regions examined. This is likely due to traditional LDDMM overfitting landmark placement inaccuracies, while GDAS finds an appropriate balance between landmark matching accuracy and shape variability. Differences show statistical significance (*P* < 0.05 in a Student's paired *t*-test) with the exception of vertices close to the head (50%: *P* = 0.4073, 80%: *P* = 0.0895).

**Figure 10:**
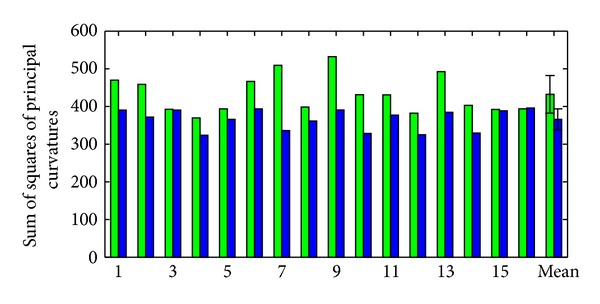
Integrated sum of squares of principal curvatures is shown for the 16 patients examined, as well as means and standard deviations. Green/left of pair: typical landmark matching; blue/right of pair: GDAS landmark matching.

To further quantify the more natural shapes produced by GDAS, we examine the curvature of the resulting segmentations. For each patient examined, the integrated sum of squares of principal curvatures is shown in Figure 10. In all but one case, the GDAS algorithm results in surfaces with less curvature. The differences are statistically significant (*P* < 0.0001 in a Student's paired *t*-test).

### 5.6. Evaluation: Simulated Data

**Figure 11:**
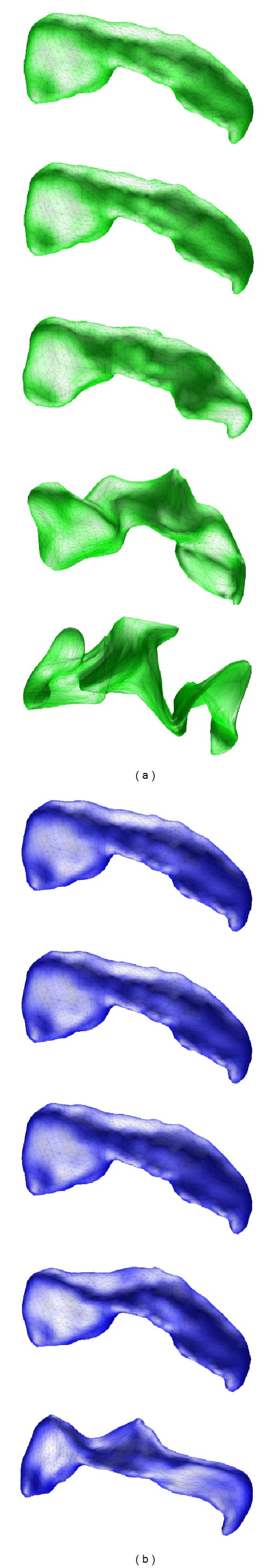
Example result from simulated data. (a) Standard landmark matching, (b) GDAS landmark matching. Landmark variance from top to bottom: 0.004475, 0.04475, 0.4475, 4.475, and 44.75.

To further quantify the performance and robustness of our landmark matching algorithm, we evaluate it using simulated data such that the gold standard segmentation can be precisely known.  Figure 11 shows example results of our landmark matching algorithms as described in Section 4.5, with traditional landmark matching shown on the left side (green) and GDAS landmark matching shown on the right side (blue). From top to bottom, the additive noise in landmark placement increases from 1/10 to 10 times that expected from voxel size in our case study (variance 0.004475, 0.04475, 0.4475, 4.475, and 44.75). At low levels of landmark uncertainty, the two algorithms give very similar results. However, as landmark uncertainty increases, the performance of GDAS exhibits a graceful decline, while that of traditional LDDMM demonstrates a precipitous drop. Note that third row gives a level of landmark uncertainty comparable to that in our case study.

**Figure 12:**
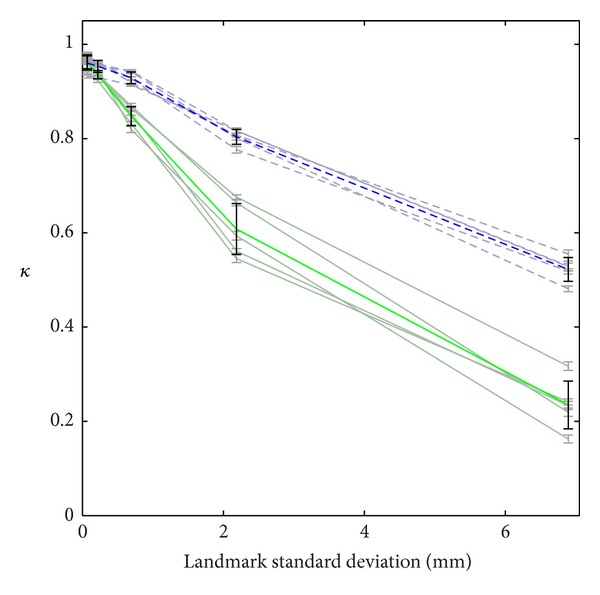
Kappa scores are shown for the 5 simulated cases (desaturated) and mean and standard deviation (saturated), as a function of landmark noise. Green/bottom: standard landmark matching; blue/top: GDAS landmark matching.

Figure 12 shows *κ* scores as a function of landmark noise for each of the five simulated cases (desaturated colors), as well as for the average performance (saturated colors). Consistent with our expectations of improved robustness, we see a much smaller variability in *κ* scores for GDAS. Furthermore, consistent with our earlier discussion of accuracy, we see poor performance of traditional LDDMM due to overfitting untrustworthy data. Note that the third data point (close to the left-hand side of the figure) corresponds to a level of landmark uncertainty comparable to that in our case study.

For the GDAS results, we can also express accuracy by measuring the error in PCA coefficients recovered by the algorithm. A natural way to do this is through the Mahalanobis distance (treating the inverse of the covariance matrix as a bilinear symmetric operator defining an inner product). Loosely, this distance is the square root of the sum of squares of differences in PCA coefficients; each being first divided by its respective standard deviation. At the five levels of landmark noise examined, the distance between the true coefficients and those recovered by GDAS (summed over the 31 coefficients) is given by 0.8100, 2.0484, 4.0477, 5.5894, and 5.7708 standard deviations. However, the lower order coefficients, which contribute more to overal shape, are recovered with more accuracy than the higher ones. The first coefficient is recovered with an error of 0.0154, 0.0259, 0.1422, 0.4338, and 0.6215 standard deviations, and the first 5 with an error of 0.0813, 0.2791, 0.5213, 1.6052, 2.0663 standard deviations.

**Figure 13:**
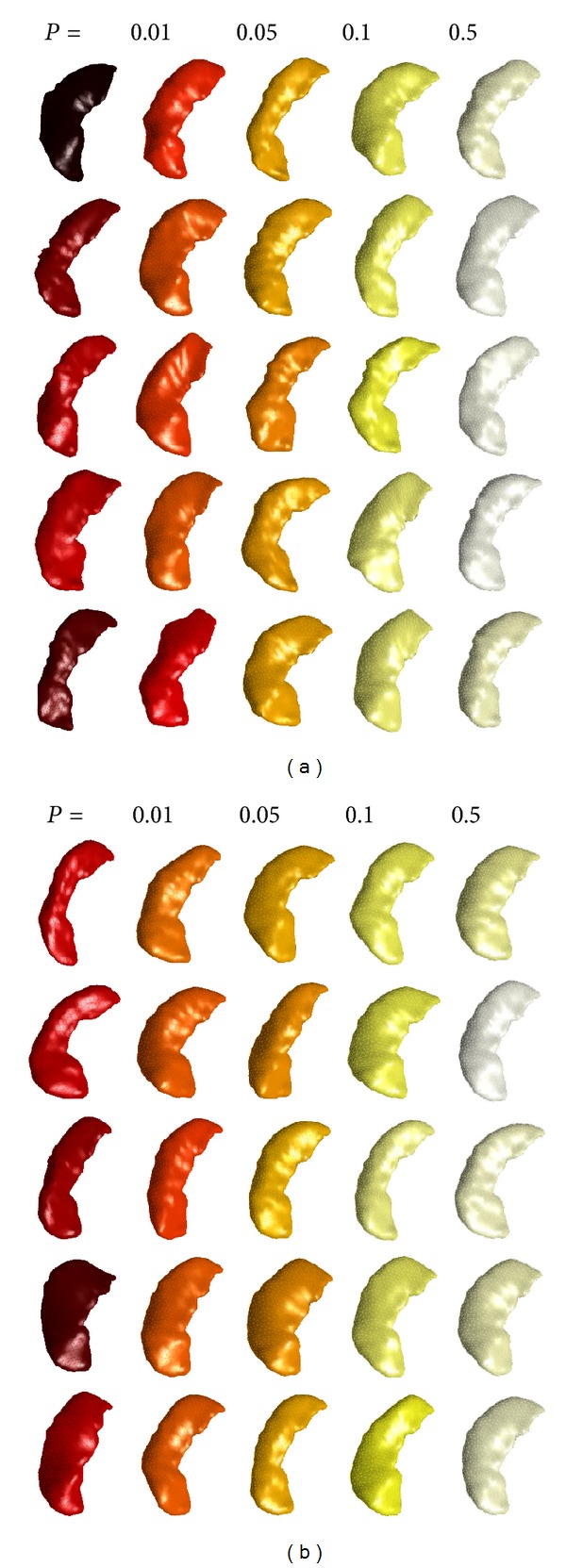
Population of left hippocampus surfaces binned by *P* value from ADNI dataset. (a) Real patient data. (b) Simulated data.

This highlights a potential future direction for the GDAS framework. We calculate the Mahalanobis distance from the origin for each of the 650 patients in our ADNI training set and use the empirical distribution to calculate *P* values. A sample of these patients is shown in Figure 13(a). Surfaces are colored by their *P* value and binned for *P* between the values {0,0.01,0.05,0.1,0.5,1}. Each column represents one bin, and five-example cases per bin are shown. It is evident that such a distance is descriptive of the naturalness of anatomical shapes, with low *P* values corresponding to unnatural shapes. Using such a tool to identify outliers for targeted quality control is the subject of future research.

An extension of this idea is the ability to generate random anatomical shapes and quantify their typicality with *P* values. Some examples are shown in Figure 13(b), with format paralleling what was discussed above. This tool demonstrates the generative nature of the GDAS framework and may prove to be useful for didactic or other purposes.

## 6. Conclusion

Volumetric segmentation has played an essential role in computer-based interpretation of medical images. There have been many approaches published to address this challenge [1, 51–58]. Most segmentation methods use a combination of shape constraint and data attachment to achieve their goals. Data attachment can be based on geometric data, gray levels, edge detection [59], or unstructured segmentation like K-Means [60] or Gaussian Mixtures [61].

In this paper, we have demonstrated applications of the GDAS framework with data attachment based on landmarks, surfaces, and likelihood ratios from grayscale values. For the case of landmark matching, we demonstrated how it could be used to remove or hasten a laborious quality control phase of large-scale neuroanatomical studies. We quantified its improvement over an existing method based on accuracy, as well as robustness. As is typical of Bayesian analysis, we originally expected to see a tradeoff between accuracy and robustness. However, our results showed improvements in both, likely due to overfitting to noisy data in the standard method, reducing accuracy.

The GDAS algorithm shows improvement over traditional landmark matching in *κ* scores and surface-to-surface distances, as compared to the gold standard segmentation. Qualitatively, improvements are particularly noticeable in the region around the hippocampus' head (where landmark placement is uncertain). The segmentations resulting from GDAS appear natural, reflecting the typicality and variability of the population from which the PCA basis was determined. This naturalness was quantified in terms of reduced curvature as compared to the traditional method and can be understood in terms of Mahalanobis distance *P* values.

We have found in large sample studies that robustness is accommodated by our GDAS methods controlled by the PCA dimensions empirically trained from samples of subcortical anatomy. In a study with over 400 hand placements of landmarks in hippocampus and amygdala, we have found that robust GDAS detects our failed landmark based mappings using *P*-values, supporting the notion that it provides direct method for quality control of large deformation mappings. Exploring this possibility will be the subject of future research.

Our method is based on the geodesically controlled diffeomorphism constraints associated with the momentum conservation law. Encoding structure via prior distributions which are empirically trained has a longstanding tradition in active shape and appearance modeling [4, 62], defined on landmark structures as well as on higher dimensional structures as proposed in [3, 63, 64]. Our principal contribution here has been to encode the diffeomorphism constraint into the standard active shape models. By incorporating the conservation law controls, we not only inherit the power of diffeomorphic transfer of the submanifold surface in the background 3D space, as has been described in [7, 8], but also obtain the metric structure property. Along the geodesic path connecting templates and targets; the metric structure of the large space is maintained.

These properties have been explicitly modelled in our own methods previously using deformable templates acted upon by diffeomorphisms and embedding them into the associated metric space structures [12, 65]. These formulations have tended to explicitly model the transformations on the entire dense background space *Ω*, working to minimize a cost function accumulated over the entire space. In the setting where the template consists of a collection of homogeneous substructures, we would expect to obtain similar formulations as described herein. In fact, Qiu and Miller [48] have used dense deformations for statistical modeling in such settings via their support on the boundaries of the cortical substructures.

## Figures and Tables

**Algorithm 1:**
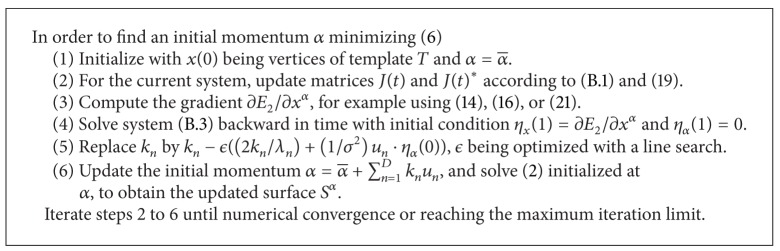
Geodesically controlled diffeomorphic segmentation algorithm.

**Algorithm 2:**
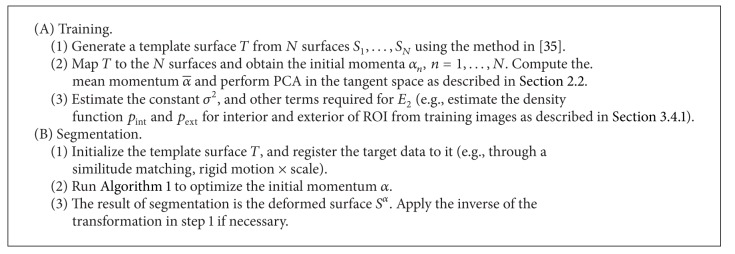
Geodesically controlled diffeomorphic active shapes.

**Table 1:** Interrater variability is examined by presenting *κ* overlap between various pairs of data (indicated in the left column).

Case	1	2	3	4	5	Average
LDDMM versus Manual Segs.	0.780	0.743	0.718	0.719	0.702	0.732
GDAS versus Manual Segs.	0.784	0.768	0.752	0.721	0.731	0.751
Manual Segs.	0.866	0.853	0.813	0.800	0.784	0.823
LDDMM versus GDAS	0.832	0.820	0.830	0.827	0.829	0.828

## Appendices

### A. Image Segmentation Energy Gradient

Theorem A.1 The gradient of (18) with respect to the *k*th deformed template vertex is given by (19).

In the proof of the theorem, we will use the following lemma (see [34] for a proof).

Lemma A.2 Let *S* be a surface and *v* a smooth function on *S* with values in ℝ^3^. For small *ϵ* > 0, let *S*_*ϵ*_ denote the surface *S* + *ϵv*_|_*S*__. Let *h* be a smooth vector field on ℝ^3^. One has

(A.1) ∂ϵ(∫SϵhTnϵdσϵ)|ϵ=0  =∫∂Sdet⁡(τ,h,v)dℓ+∫S(∇·h)vTn dσ,

where *τ* is the positively oriented tangent to the boundary of *S*, *n*_*ϵ*_ and *σ*_*ϵ*_, respectively, denote the unit normal and the area form on *S*_*ϵ*_, and ∇·*h* is the divergence of *h*.

Proof of Theorem A.1
First notice that by adding and subtracting ∫_*x*∈int⁡(*S*)_log⁡  [*p*_int⁡_(*I*(*x*))]*dx*, we can rewrite the energy as

(A.2) $$\begin{array}{c} E_{2}=\int_{x\in \mathrm{int}(S)}g\left(x\right)dx+\text{cst}, \end{array}$$

where the constant, cst, has gradient zero. Define surface faces and normals as in Section 3.2.1. Note that the unit normal on a face is *n*_*f*_^*α*^ = *N*_*f*_^*α*^/|*N*_*f*_^*α*^|, the area of face *f* being |*N*_*f*_^*α*^|. A generic point in face *f* takes the form

(A.3) $$\begin{array}{c} y=\lambda_{1}x_{f,1}^{\alpha }+\lambda_{2}x_{f,2}^{\alpha }+\lambda_{3}x_{f,3}^{\alpha }, \end{array}$$

with *λ*_1_, *λ*_2_, *λ*_3_ ≥ 0 and *λ*_1_ + *λ*_2_ + *λ*_3_ = 1. These coefficients are explicitly given by

(A.4) $$\begin{array}{c} \lambda_{j}N_{f}=\frac{1}{2}\left(m_{f,j}^{\alpha }-y\right)\times e_{f,j}^{\alpha }. \end{array}$$

A variation *x*_*k*_^*α*^ ↦ *x*_*k*_^*α*^ + *ϵη*_*k*_ for *k* = 1,…, *L* implies for such a *y* the transformation *y* + *ϵv*(*y*) with

(A.5) $$\begin{array}{c} v\left(y\right)=\lambda_{1}\eta_{f,1}+\lambda_{2}\eta_{f,2}+\lambda_{3}\eta_{f,3}. \end{array}$$

The mapping *v* which is defined this way is a continuous vector field on *S* and smooth (linear) over each face. Introduce a function *h* such that ∇·*h* = *g* (such an *h* always exists). By Stokes theorem, we have

(A.6) $$\begin{array}{c} E_{2}=\int_{S}\left(h^{T}n\right)d\sigma +\text{cst}. \end{array}$$

We can decompose this integral over all faces and apply (A.1) in Lemma A.2 to each face. Summing the result over the faces and noting that the boundary terms cancel (because the tangents of two neighbor faces are in opposite directions), we find

(A.7) $$\begin{array}{c} {\left(\frac{dE_{2}}{dx^{\alpha }}\right)}^{T}\eta =\sum_{f\in F}\int_{f}g\;\left(v\cdot n_{f}\right)d\sigma_{f}. \end{array}$$

Replacing *v* by its expression and reordering the sum over the vertices yield

(A.8) (dE2dxα)Tη =∑k=1LηkT(∑f:xkα=xf,iα12|Nf|∫fg(y)(mf,iα−y)×ef,iαdσf).

Equation (19) follows, completing the proof.

### B. Adjoint Method

We need the following definitions, associated to the variation of (2) and its transpose. Applying an infinitesimal variation *α* → *α* + *δα* in the initial condition induces infinitesimal variations *a* + *δa* and *x* + *δx*, and the pair (*δx*, *δa*) obeys the following differential system, that can be obtained from a formal variation of (2):

(B.1) dδxk(t)dt=∑l=1Lγklδal+2γkl′al(xk−xl)·(δxk−δxl),dδak(t)dt=∑l=1L−2γkl′(al·δak+δal·ak)(xk−xl) −2∑l=1Lγkl′(al·ak)(δxk−δxl) −4∑l=1Lγkl′′(al·ak)(xk−xl)  ×((xk−xl)·(δxk−δxl)),

with *γ*_*kl*_′′ = *γ*′′(||*x*_*k*_ − *x*_*l*_||^2^) (recall that *γ*_*kl*_, *γ*_*kl*_′ are short for *γ*(||*x*_*k*_ − *x*_*l*_||^2^) and *γ*′(||*x*_*k*_ − *x*_*l*_||^2^)).

This linear system can be rewritten in matrix form:

(B.2) $$\begin{array}{c} \frac{\text{d}}{\text{d}t}\left(\begin{array}{c} \delta x\\ \delta \alpha \end{array}\right)=J\left(t\right)\left(\begin{array}{c} \delta x\\ \delta \alpha \end{array}\right). \end{array}$$

We let *ζ*(*x* ^0^, *α*, *ρ*) denote the solution at *t* = 1 of (B.1) initialized at *δx*(0) = 0 and *δα*(0) = *ρ*, solved along with (2) initialized with (*x* ^0^, *α*). We define the dual variation *ζ**(*x* ^0^, *α*, *ξ*) as follows. First, solve (2) with initial conditions *x*(0) = *x* ^0^ and *a*(0) = *α*. Then, solve

(B.3) $$\begin{array}{c} \frac{\text{d}}{\text{d}t}\left(\begin{array}{c} \eta_{x}\\ \eta_{\alpha } \end{array}\right)=-{J\left(t\right)}^{*}\left(\begin{array}{c} \eta_{x}\\ \eta_{\alpha } \end{array}\right), \end{array}$$

from time *t* = 1 to time *t* = 0 with *η*_*x*_(1) = *ξ* and *η*_*α*_(1) = 0, and set

(B.4) $$\begin{array}{c} \zeta^{*}\left(x^{0},\alpha ,\xi \right)=\eta_{\alpha }\left(0\right). \end{array}$$

We state without proof the following lemma, which is a consequence of the theory of linear differential systems (cf. [34, 35] for a proof).

Lemma A.3 For dynamic system (2), let *dx* ^*α*^/*dα* denote the Jacobian matrix of the nonlinear transformation *α* ↦ *x* ^*α*^, represented as a 3*L* by 3*L* matrix. One has, for a momentum vector *ρ*,

(B.5) $$\begin{array}{c} \left(\frac{dx^{\alpha }}{d\alpha }\right)\rho =\zeta \left(x^{0},\alpha ,\rho \right), \end{array}$$

and, for a vector *ξ* ∈ ℝ^3*L*^,

(B.6) $$\begin{array}{c} {\left(\frac{dx^{\alpha }}{d\alpha }\right)}^{T}\xi =\zeta^{*}\left(x^{0},\alpha ,\xi \right). \end{array}$$

In our application, setting *ξ* = ∂*E*_2_/∂*x* ^*α*^, we retreive

(B.7) (∂E2∂α)=(∂xα∂α)T(∂E2∂xα)=ζ∗(x0,α,∂E2∂xα),

which is used in calculating the energy gradient.
