# Corrigendum to “Robust Diffeomorphic Mapping via Geodesically Controlled Active Shapes”

**DOI:** 10.1155/2021/9780202

**Published:** 2021-05-26

**Authors:** Daniel J. Tward, Jun Ma, Michael I. Miller, Laurent Younes

**Affiliations:** ^1^Department of Biomedical Engineering, Johns Hopkins University, Baltimore, MD 21218, USA; ^2^Siemens Healthcare, Hoffman Estates, Chicago, IL 60192, USA; ^3^Center for Imaging Science, Johns Hopkins University, Baltimore, MD 21218, USA

In the article titled “Robust Diffeomorphic Mapping via Geodesically Controlled Active Shapes” [1], the authors did not originally refer to funding received from AnatomyWorks LLC and therefore wish to make the following addition to the acknowledgments section:

“Funding for the study was provided by the NIH under a license agreement between AnatomyWorks LLC and the Johns Hopkins University. Dr. Michael I. Miller and the University are entitled to royalty distributions related to technology described in the study. Dr. Miller is a founder of and holds equity in AnatomyWorks LLC. This arrangement has been reviewed and approved by the Johns Hopkins University in accordance with its conflict of interest policies.”
